# Supplementary material for: StableMARK-decorated microtubules in cells have expanded lattices
Source: J Cell Biol. 2024 Oct 10;224(1):e202206143. doi: 10.1083/jcb.202206143 (PMC11471893; doi:10.1083/jcb.202206143)
Supplement: Table S1 — lists previously reported microtubule lattice spacings with respect to nucleotide state, Taxol treatment, and microtubule-binding proteins. [file JCB_202206143_TableS1.docx]

Supplementary table 1. **Previously reported microtubule lattice spacings with respect to nucleotide state, Taxol treatment and MT-binding proteins.**

| Microtubule component | References |
| --- | --- |
| *In vitro* GDP-MT | (Estevez-Gallego et al., 2020; Hyman et al., 1995; LaFrance et al., 2022; Rai et al., 2020; Vale et al., 1994; Zhang et al., 2018) |
| *In vitro* Taxol-MT | (Alushin et al., 2014; Estevez-Gallego et al., 2020; Kellogg et al., 2017; Rai et al., 2020; Vale et al., 1994) |
| *In vitro* GMPCPP-MT | (Estevez-Gallego et al., 2020; Hyman et al., 1995; Kellogg et al., 2017; LaFrance et al., 2022; Vale et al., 1994; Zhang et al., 2018) |
| *In situ* GDP-MT/LRRK2 | (Watanabe et al., 2020) |
| *In vitro* DCX | (Bechstedt and Brouhard, 2012; Manka and Moores, 2018b) |
| *In vitro* Kinesin-1 | (Peet et al., 2018; Shima et al., 2018) |
| *In vitro* Tau/MAP2/MAP4 | (Kellogg et al., 2018; Siahaan et al., 2022) |
| *In vitro* EB3 | (LaFrance et al., 2022; Zhang et al., 2015; Zhang et al., 2018) |
| *In vitro* TPX2 | (Zhang et al., 2018; Zhang et al., 2017) |
| *In vitro* Kinesin-3 | (Guedes-Dias et al., 2019) |
| *In vitro* MAP7 | (Ferro et al., 2022) |
