# Supplementary material for: StableMARK-decorated microtubules in cells have expanded lattices
Source: J Cell Biol. 2024 Oct 10;224(1):e202206143. doi: 10.1083/jcb.202206143 (PMC11471893; doi:10.1083/jcb.202206143)
Supplement: Table S2 — shows cryo-ET data collection parameters. [file JCB_202206143_TableS2.docx]

*Supplementary table 2.* ***Cryo-ET data collection parameters.***

|  | *Dataset 1* | *Dataset 2* | *Dataset 3* | *Dataset 4* | *Dataset 5* | *Dataset 6* | *Dataset 7* | *Dataset 8* |
| --- | --- | --- | --- | --- | --- | --- | --- | --- |
| *Sample type* | *Untreated* | *In situ Taxol* | *Stable-MARK* | *In vitro*  *Taxol 1* | *In vitro GMPCPP* | *In vitro Dynamic* | *In vitro Taxol Unlabelled* | *In vitro GMPCPP*  *Unlabelled* |
| *Magnification* | *63.000* | *63.000 or 42.000* | *63.000 or 42.000* | *63.000* | *63.000* | *63.000* | *63.000* | *63.000* |
| *Voltage (kV)* | *200* | *200 or 300* | *200 or 300* | *200* | *200* | *200* | *200* | *200* |
| *Dose (e-/Å^2^)* | *<100* | *<100* | *<100* | *<100* | *<100* | *<100* | *<100* | *<100* |
| *Defocus (µm)* | *2-3* | *2.3-3.2* | *1.9-3* | *2-3* | *1.4-2.5* | *1.3-1.7* | *1.5-2.6* | *1.5-3* |
| *Pixel size (Å)* | *2.17* | *2.17* | *2.17* | *2.17* | *2.17* | *2.17* | *2.17* | *2.17* |
| *Increment (⁰)* | *2* | *3* | *2 or 3* | *3* | *3* | *3* | *3* | *3* |
| *Tilt range* (⁰)* | *+/-55* | *+/-54* | *+/-54* | *+/- 60* | *+/- 60* | *+/- 54* | *+/- 60* | *+/- 60* |
| *# of tilt series* | *12* | *6* | *25* | *3* | *14* | *6* | *2* | *2* |

**Tilt range here is corrected for lamella pre-tilt, e.g. -54⁰ to +54⁰ could mean a stage tilt of be -63⁰ to +45⁰, +63⁰ to -49⁰, -65⁰ to + 43⁰ etc. This depends on lamella angle (mostly 9-11⁰) and orientation.*
